# Characteristics of Patients with Pulmonary Arterial Hypertension Receiving Selexipag in the SPHERE Registry by Race and Ethnicity

**DOI:** 10.1007/s40615-025-02579-3

**Published:** 2025-08-04

**Authors:** Harrison W. Farber, Murali M. Chakinala, Anna R. Hemnes, Kelly M. Chin, Kristin B. Highland, Vallerie V. McLaughlin, Lana D. Melendres-Groves, Michelle Cho, Gurinderpal Doad, Elham Fatehi, Michelle Han, Mohammad Rahman, Paul Strachan, Tobore Tobore, Nick H. Kim

**Affiliations:** 1 Tufts Medical Center, 800 Washington Street, #257 (Tupper 3), Boston, MA 02111 USA; 2 Washington University School of Medicine, St. Louis, MO USA; 3 Vanderbilt University Medical Center, Nashville, TN USA; 4 University of Texas, Southwestern Medical Center, Dallas, TX USA; 5 Cleveland Clinic, Cleveland, OH USA; 6 University of Michigan Medical Center, Ann Arbor, MI USA; 7 University of New Mexico, Albuquerque, NM USA; 8 Johnson & Johnson, Titusville, NJ USA; 9 University of California San Diego, La Jolla, San Diego, CA USA

**Keywords:** Ethnicity, Hospitalization, Prescribing patterns, Pulmonary arterial hypertension, Race, Selexipag

## Abstract

**Introduction:**

Racial/ethnic minority populations in the US have a high burden of pulmonary arterial hypertension (PAH).

**Objective:**

To evaluate demographics, disease characteristics, prescribing patterns, hospitalization, and survival in racial/ethnic groups in the SPHERE registry.

**Methods:**

SPHERE was a US, multicenter, prospective, observational registry of adults prescribed selexipag in clinical practice (November 2016–March 2020). Follow-up was ≤ 18 months, with data collected at routine quarterly visits.

**Results:**

There were 759 patients with PAH: 549 (72.3%) non-Hispanic White; 117 (15.4%) Black/African American; 45 (5.9%) Hispanic; and 48 (6.3%) other/unknown race/ethnicity. Overall, 50.6% of participants had idiopathic and 27.0% had connective tissue disorder-associated PAH. Median age at diagnosis in non-Hispanic White, Black/African American, and Hispanic groups was 57, 56, and 43 years, and at selexipag initiation was 62, 60, and 51 years, respectively. Hispanic participants had less severe symptoms at enrollment: 40.0% had World Health Organization functional class III versus 53.4% and 50.4% of non-Hispanic White and Black/African American participants, respectively. Comorbidities were high, with some differences between groups. No notable differences existed between groups in selexipag dose or PAH-specific therapy; overall, 30.8% received monotherapy, 55.6% dual therapy, and 8.4% triple therapy before selexipag initiation. Discontinuations resulting from selexipag-related adverse events in any group were few (2.2%–7.7%). All-cause hospitalization and survival were similar across groups.

**Conclusion:**

Selexipag was prescribed as part of combination therapy for PAH regardless of differing demographics and clinical characteristics across racial/ethnic groups, with no differences in selexipag discontinuation, hospitalization, and survival.

**Supplementary Information:**

The online version contains supplementary material available at https://doi.org/10.1007/s40615-025-02579-3.

## Introduction

Pulmonary arterial hypertension (PAH) encompasses a heterogeneous group of disorders characterized by pulmonary vascular remodeling, elevation of pulmonary arterial pressure, right ventricular dysfunction and failure, and if left untreated, premature death. Approximately 1,000 new cases of PAH are diagnosed in the United States each year [1]. The cause may be genetic, drug/toxin-induced, or associated with an underlying condition, such as connective tissue disease (CTD), congenital heart disease (CHD), portal hypertension, or human immunodeficiency virus [2]. In most cases, no specific cause is identified and patients are classified as having idiopathic PAH. Prognoses can vary according to the underlying etiology.

Selexipag is an oral, selective IP prostacyclin receptor agonist approved for use in patients with PAH in the United States to delay disease progression and reduce the risk of hospitalization [3]. SPHERE (SelexiPag: tHe usErs dRug rEgistry) was established to characterize the clinical features, dosing regimens, and outcomes from a representative range of US patients receiving selexipag in routine clinic settings [4, 5]. We previously reported the first US-based real-world outcomes data for selexipag from SPHERE [4, 5], confirming that selexipag in clinical practice was similar to that seen in the randomized trial [6].

Racial/ethnic minority populations have an increased burden of PAH and its associated conditions, yet they remain under-represented in clinical trials [7, 8]. Real-world evidence illustrates variation in the prevalence of PAH subtypes according to race and ethnicity [9, 10]. For example, Black/African American individuals are more likely to develop PAH related to CTD, while CHD-associated PAH is more common in Hispanic individuals [9, 10]. While there is evidence that race and ethnicity may affect clinical outcomes [11, 12], social determinants of health, including access to care and socioeconomic status, likely also play a role [7, 11]. As the SPHERE registry includes large numbers of racial/ethnic minority populations, it provides an opportunity to examine differences in patient demographics, disease characteristics, prescribing patterns, and patient outcomes for different racial/ethnic groups receiving selexipag in the United States. The information generated might help to guide treatment decisions in populations that historically have been under-represented in clinical trials.

## Methods

The methods of the SPHERE study (NCT03278002) have been published in detail [4, 5]. For the current analysis, data from patients with PAH were analyzed descriptively by race/ethnicity.

### Study Design

In brief, SPHERE was a US-based, multicenter, prospective, observational registry that enrolled adults who were prescribed selexipag in clinical practice between November 2016 and March 2020. Participants were prospectively followed for up to 18 months, and data were collected at quarterly routine clinic visits using electronic case report forms. There were no study-mandated visits or procedures.

### Participants

Eligible participants were aged ≥ 18 years at selexipag treatment initiation. Due to the real-world nature of the study, some patients with World Health Organization (WHO) Group 2 through Group 5 pulmonary hypertension were enrolled (i.e., non-PAH); however, the present analysis includes only patients with PAH. Participants were categorized as either newly initiated on selexipag (i.e., starting selexipag ≤ 60 days before enrollment) or previously initiated on selexipag (i.e., starting selexipag > 60 days before enrollment). Individuals who discontinued selexipag before enrollment or who had been previously exposed to selexipag during a clinical trial were excluded. For newly initiated patients, baseline assessments were defined as the first available measurement between the first selexipag dose and enrollment; for previously initiated patients, they were defined as the closest measurement performed around the time of the first selexipag dose.

### Treatment

Selexipag was initiated and dosed per routine clinical practice. The recommended selexipag starting dose in the United States is 200 µg twice daily, usually titrated weekly in increments of 200 µg twice daily to an individualized dose (based on tolerability), or a maximum dose of 1,600 µg twice daily [3]. In SPHERE, the titration phase was defined as the period between selexipag initiation and the highest dose reached within 6 months. The individualized dose was the first dose post-titration maintained for ≥ 14 days without dose interruption/change.

### Data Collection

Collected data included demographics, medical history, etiology of PAH, functional status (including New York Heart Association/WHO functional class [FC], 6-min walk distance [6MWD], Borg dyspnea index score, and N-terminal pro-B-type natriuretic peptide [NT-proBNP] level), PAH risk score (per the Registry to Evaluate Early and Long-term PAH Disease Management [REVEAL] risk calculator, REVEAL 2.0 [13], and its abridged version, REVEAL Lite 2 [14]), prior and concomitant PAH medication, selexipag dosing, and patient outcomes (including time to first hospitalization, safety, and discontinuation due to adverse events [AEs] or progression). Race/ethnicity was self-reported by patients.

### Statistical Analysis

Data were analyzed descriptively for the overall population of patients with PAH (i.e., WHO Group 1 pulmonary hypertension), and according to race/ethnicity (non-Hispanic White, Black/African American, Hispanic, and other race/ethnicity). Due to the small number of patients of other race/ethnicity enrolled, analyses focused on non-Hispanic White, Black/African American, and Hispanic cohorts, although data for the Other cohort are shown in the tables and figures. Kaplan–Meier analyses (with 95% confidence interval [CI]) were used to describe time to first all-cause hospitalization. Hazard ratios with 95% CIs for survival were calculated for Black/African American and Hispanic participants relative to non-Hispanic White participants. There was no imputation for missing observations.

### Ethics

Institutional review board and/or ethical committee approval was granted at each participating site in accordance with local and national regulations/policies, and all patients provided written informed consent.

## Results

### Patient Demographics

#### Race/Ethnicity and Sex

Of the 829 patients with pulmonary hypertension enrolled in SPHERE at approximately 80 centers, 759 (91.6%) were diagnosed with PAH and included in the analysis. Of these, 549 (72.3%) were non-Hispanic White, 117 (15.4%) Black/African American, 45 (5.9%) Hispanic, and 48 (6.3%) were of other race/ethnicity (26 [3.4%] Asian, 3 [0.4%] Native Hawaiian/Pacific Islander, 2 [0.3%] American Indian/Alaska Native, 10 [1.3%] another race/ethnicity, and 7 [0.9%] unknown race/ethnicity). Most participants were female (76.5%), with the highest proportion among Black/African American (86.3%) and Hispanic (84.4%) groups, compared with non-Hispanic Whites (72.7%) (Table 1).

**Table 1:** Participant demographics and disease characteristics, according to race/ethnicity

Parameter	Non-Hispanic White (*n* = 549)	Black/African American (*n* = 117)	Hispanic (*n* = 45)	Other^a^ (*n* = 48)	Overall (*N* = 759)
Age (years) at PAH diagnosis, median (IQR)	*n* = 549	*n* = 117	*n* = 45	*n* = 48	*N* = 759
	57 (45–67)	56 (45–61)	43 (37–54)	41 (31–50)	55 (43–65)
Age (years) at selexipag initiation, median (IQR)	*n* = 549	*n* = 117	*n* = 45	*n* = 48	*N* = 759
	62 (51–71)	60 (51–65)	51 (46–61)	46 (35–59)	61 (49–69)
Time (years) from PAH diagnosis to selexipag initiation, median (IQR)	*n* = 549	*n* = 117	*n* = 45	*n* = 48	*N* = 759
	2.6 (0.9–6.3)	3.0 (1.4–7.1)	4.0 (1.1–10.3)	3.3 (1.6–10.3)	2.7 (1.1–6.9)
Female, *n* (%)	*n* = 549	*n* = 117	*n* = 45	*n* = 48	*N* = 759
	399 (72.7)	101 (86.3)	38 (84.4)	43 (89.6)	581 (76.5)
BMI (kg/m^2^) at selexipag initiation, median (IQR)	*n* = 519	*n* = 111	*n* = 43	*n* = 46	*N* = 719
	28.7 (24.7–34.1)	29.7 (23.3–35.4)	28.3 (25.4–32.3)	26.6 (21.4–30.3)	28.5 (24.5–34.4)
PAH at selexipag initiation, *n* (%)	*n* = 549	*n* = 117	*n* = 45	*n* = 48	*N* = 759
Newly initiated	87 (15.8)	13 (11.1)	4 (8.9)	3 (6.3)	107 (14.1)
Previously initiated	462 (84.2)	104 (88.9)	41 (91.1)	45 (93.8)	652 (85.9)
WHO classification of Group 1 PAH at diagnosis, *n* (%)	*n* = 549	*n* = 117	*n* = 45^b^	*n* = 48	*N* = 759
Idiopathic	282 (51.4)	65 (55.6)	17 (37.8)	20 (41.7)	384 (50.6)
Heritable	12 (2.2)	2 (1.7)	0	1 (2.1)	15 (2.0)
Drug/toxin-induced	36 (6.6)	3 (2.6)	4 (8.9)	4 (8.3)	47 (6.2)
Associated					
Connective tissue disease	144 (26.2)	38 (32.5)	15 (33.3)	8 (16.7)	205 (27.0)
Congenital heart disease	31 (5.6)	3 (2.6)	3 (6.7)	3 (6.3)	40 (5.3)
Portal hypertension	18 (3.3)	0	4 (8.9)	2 (4.2)	24 (3.2)
HIV infection	3 (0.5)	4 (3.4)	0	1 (2.1)	8 (1.1)
PVOD/PCH	2 (0.4)	0	1 (2.2)	0	3 (0.4)
Other	18 (3.3)	2 (1.7)	2 (4.4)	8 (16.7)	29 (3.8)
Unknown	3 (0.5)	0	0	1 (2.1)	4 (0.5)
6MWD (m), median (IQR)	*n* = 452	*n* = 90	*n* = 31	*n* = 41	*N* = 614
	320.0 (222.0–403.1)	278.0 (183.0–372.0)	368.8 (259.0–430.0)	395.0 (301.0–465.0)	320.0 (222.0–405.0)
Borg dyspnea index score, median (IQR)	*n* = 412	*n* = 88	*n* = 29	*n* = 39	*N* = 568
	3.0 (2.0–4.0)	3.0 (1.0–5.0)	3.0 (0.5–4.0)	2.0 (1.0–5.0)	3.0 (2.0–4.5)
NT-proBNP (ng/L), median (IQR)	*n* = 173	*n* = 35	*n* = 6	*n* = 15	*N* = 229
	687.0 (183.0–1707.0)	448.0 (140.0–2970.0)	269.0 (69.0–595.0)	339.0 (166.0–596.0)	595.0 (168.0–1651.0)

6MWD, 6-min walk distance; *BMI* body mass index, *HIV* human immunodeficiency virus, *IQR* interquartile range, *NT-proBNP* N-terminal pro-B-type natriuretic peptide, *PAH* pulmonary arterial hypertension, *PCH* pulmonary capillary hemangiomatosis, *PVOD* pulmonary veno-occlusive disease, *WHO* World Health Organization

^a^ Other group: Asian (*n* = 26), Native Hawaiian/Pacific Islander (*n* = 3), American Indian/Alaska Native (*n* = 2), another race/ethnicity (*n* = 10), and unknown race/ethnicity (*n* = 7)

^b^ One patient identified as connective tissue disease and portopulmonary hypertension

Variability in race/ethnicity was observed across treatment site regions. The highest proportion of non-Hispanic White participants was in the Midwest (80.9%), the highest proportion of Black/African American participants was in the Southeast (25.9%), and the highest proportion of Hispanic participants was in the Southwest (20.0%) (Fig. S1 in Online Resource 1).

#### PAH Subtypes

Overall, 50.6% of participants had idiopathic PAH and 27.0% had CTD-associated PAH (Table 1). Black/African American and Hispanic participants had the highest rates of CTD-associated PAH (32.5% and 33.3%, respectively). Rates of drug/toxin-induced PAH (8.9%), CHD-associated PAH (6.7%), and portal hypertension (8.9%) were highest among the Hispanic population. Hispanic participants had fewer idiopathic cases (37.8%) compared with non-Hispanic White (51.4%) and Black/African American (55.6%) participants (Table 1).

#### Age at Diagnosis/Selexipag Initiation

Median age at PAH diagnosis and selexipag initiation was 55 and 61 years, respectively, for the overall PAH population, with a median time from PAH diagnosis to selexipag initiation of 2.7 years (Table 1). Hispanic participants had the lowest median age at PAH diagnosis and selexipag initiation (43 and 51 years, respectively) and the longest median time from PAH diagnosis to selexipag initiation (4.0 years). Non-Hispanic White participants were, by contrast, older at PAH diagnosis and selexipag initiation (57 and 62 years, respectively), and had the shortest time from diagnosis to selexipag initiation (2.6 years).

### Disease Characteristics

**Fig. 1:**
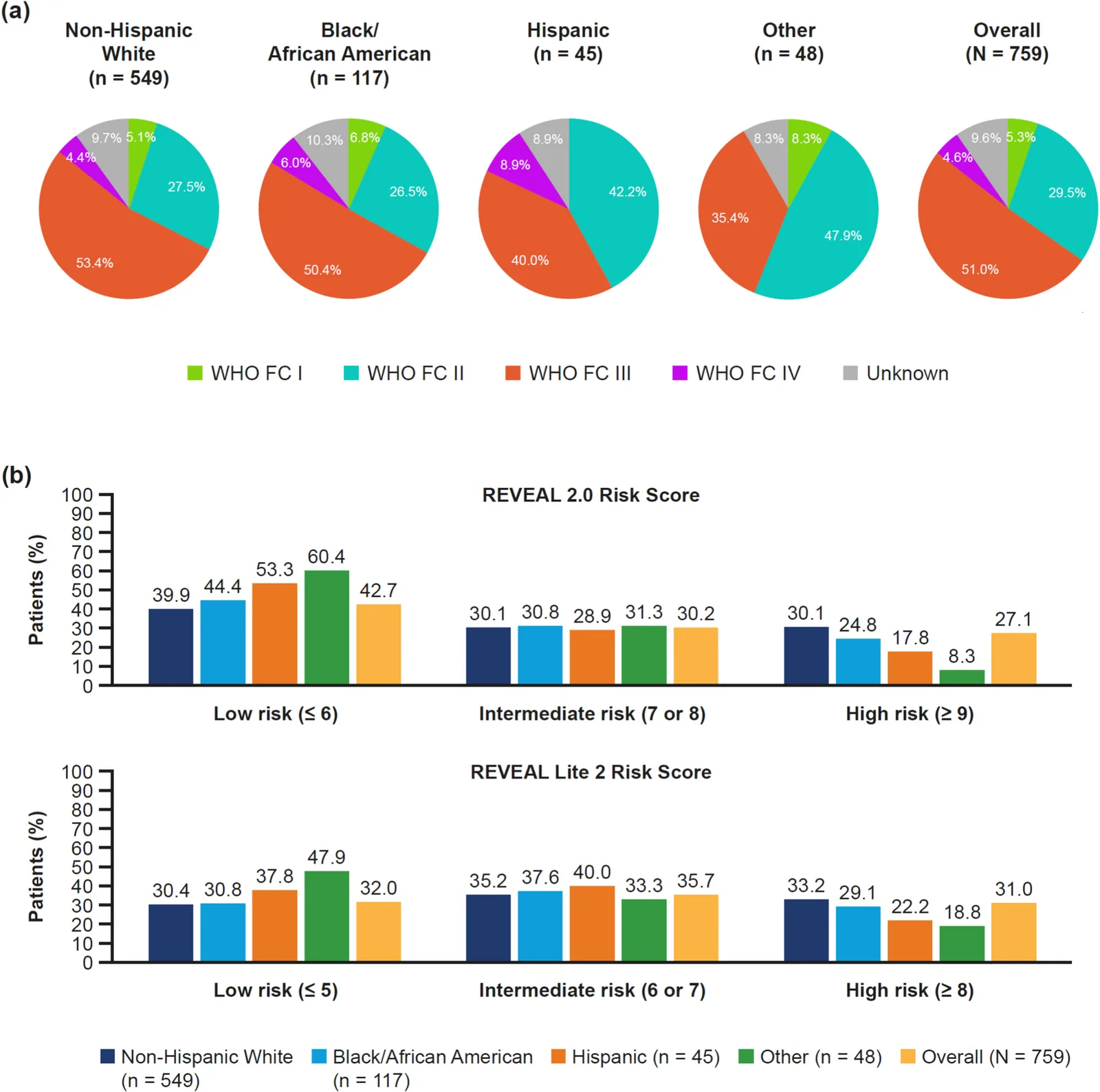
WHO functional class (**a**) and risk assessment based on REVEAL 2.0/REVEAL Lite 2 (**b**) at selexipag initiation in patients with PAH, according to race/ethnicity. Percentages may not add up to 100 due to rounding. PAH, pulmonary arterial hypertension. REVEAL, Registry to Evaluate Early and Long-term PAH Disease Management; WHO FC, World Health Organization functional class

Of the 759 patients with PAH, the majority (51.0%) were WHO FC III at selexipag initiation. There was a lower proportion of WHO FC III among Hispanic participants (40.0%) than non-Hispanic White (53.4%) or Black/African American (50.4%) participants (Fig. 1a). REVEAL 2.0/REVEAL Lite 2 baseline risk scores also suggested lower 1-year mortality risk in Hispanic versus non-Hispanic White or Black/African American participants (Fig. 1b). These results were reflected in a higher median 6MWD and lower NT-proBNP level among Hispanic participants (368.8 m and 269.0 ng/L) compared with Black/African American (278.0 m and 448.0 ng/L) and non-Hispanic White (320.0 m and 687.0 ng/L) participants at selexipag initiation (Table 1). By contrast, Borg dyspnea index scores for the three racial/ethnic groups did not differ. Taken together, these data suggest that selexipag is started earlier in the disease course (when disease is less severe) in Hispanic patients compared with other groups.

**Fig. 2:**
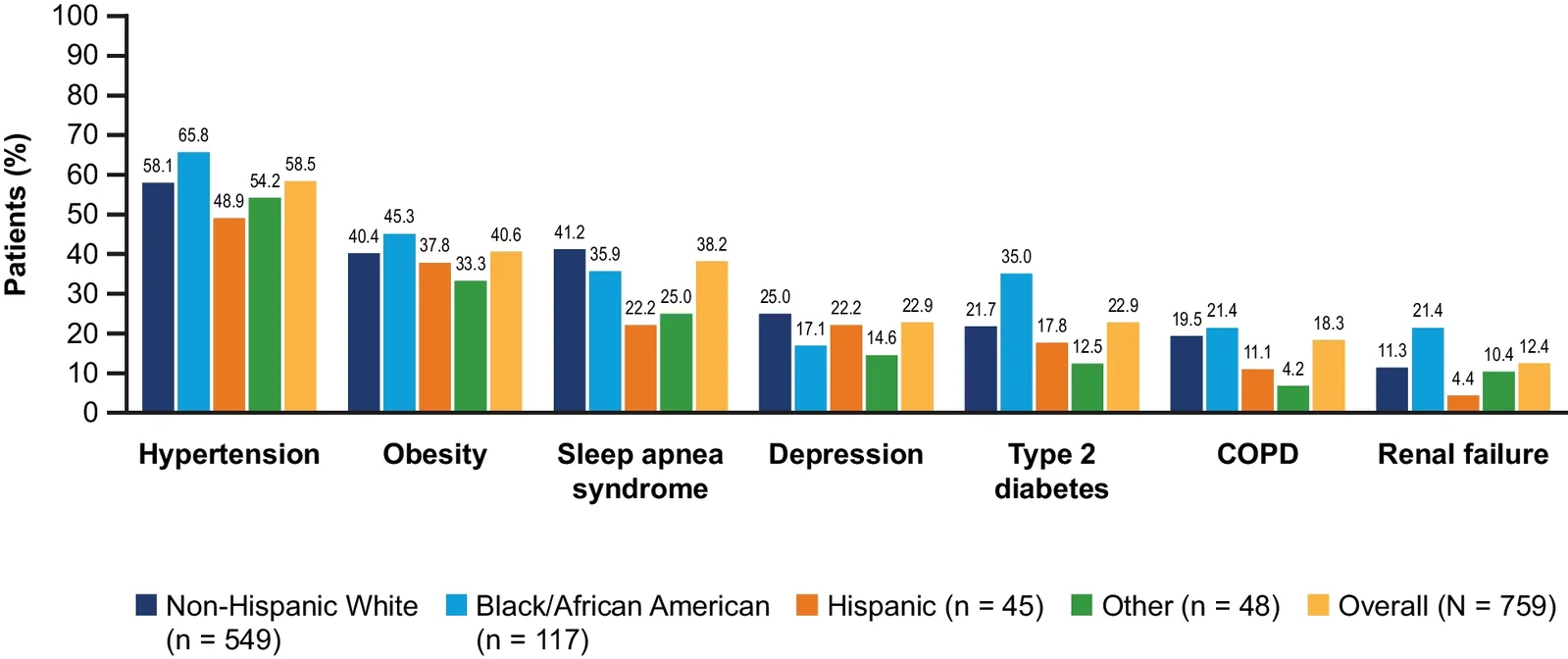
Comorbidities occurring in > 20% of patients with PAH at selexipag initiation, according to race/ethnicity. COPD, chronic obstructive pulmonary disease; PAH, pulmonary arterial hypertension

Rates of self-reported comorbid disorders were high, including hypertension (58.5%), obesity (40.6%), sleep apnea syndrome (38.2%), depression (22.9%), type 2 diabetes (22.9%), chronic obstructive pulmonary disorder (18.3%), and renal failure (12.4%) (Fig. 2). There were some differences between the racial/ethnic groups: Black/African American participants had the highest rates of renal failure (21.4%), type 2 diabetes (35.0%), and hypertension (65.8%), while Hispanic participants had the lowest rates of these conditions (4.4%, 17.8%, and 48.9%, respectively).

### Concomitant PAH-Specific Therapy at Selexipag Initiation

**Fig. 3:**
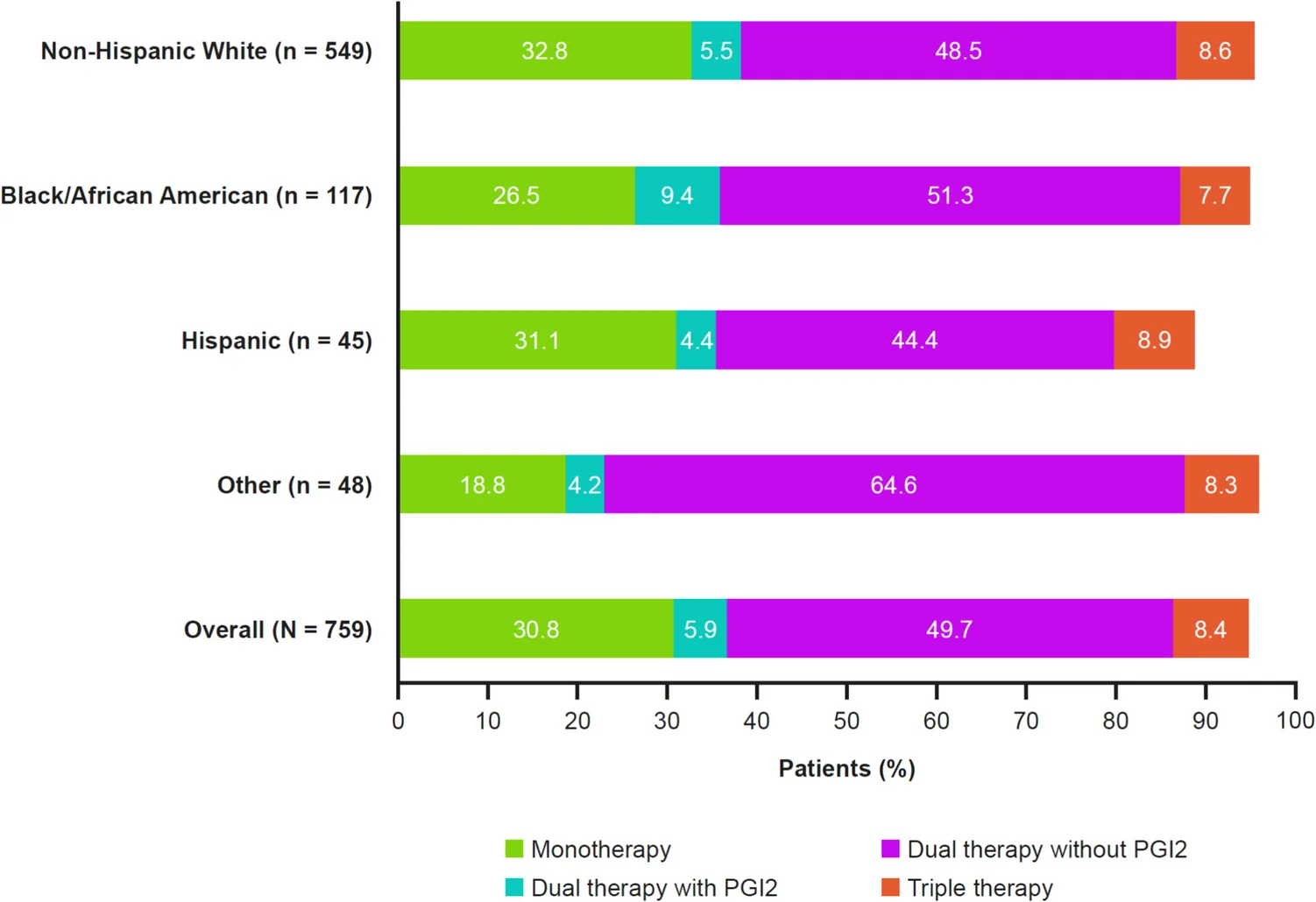
Number of concomitant PAH therapies at selexipag initiation by race/ethnicity. Concomitant PAH therapies include calcium channel blockers, endothelin receptor antagonists, phosphodiesterase-5 inhibitors, prostaglandin I2, and soluble guanylate cyclase stimulators. PAH, pulmonary arterial hypertension; PGI2, prostaglandin I2

At the time of selexipag initiation, 94.1% of study participants were taking concomitant medications for PAH, with the lowest rate among Hispanic participants (88.9%). Approximately one-third (30.8%) of all participants were receiving monotherapy, half (55.6%) were receiving dual therapy, and 8.4% were receiving triple therapy (including endothelin receptor antagonist, phosphodiesterase-5 inhibitor, prostacyclin, and soluble guanylate cyclase stimulator) (Fig. 3 and Table 2). There were no notable differences between racial/ethnic groups.

**Table 2:** Concomitant PAH therapies at selexipag initiation, according to race/ethnicity

PAH Therapy, *n* (%)	Non-Hispanic White (*n* = 549)	Black/African American (*n* = 117)	Hispanic (*n* = 45)	Other^a^ (*n* = 48)	Overall (*N* = 759)
Monotherapy	180 (32.8)	31 (26.5)	14 (31.1)	9 (18.8)	234 (30.8)
ERA	55 (10.0)	11 (9.4)	4 (8.9)	3 (6.3)	73 (9.6)
PDE5i	101 (18.4)	18 (15.4)	6 (13.3)	6 (12.5)	131 (17.3)
PGI2	8 (1.5)	2 (1.7)	3 (6.7)	0	13 (1.7)
sGC	16 (2.9)	0	1 (2.2)	0	17 (2.2)
Dual therapy with PGI2	30 (5.5)	11 (9.4)	2 (4.4)	2 (4.2)	45 (5.9)
ERA and PGI2	16 (2.9)	6 (5.1)	2 (4.4)	1 (2.1)	25 (3.3)
PDE5i and PGI2	13 (2.4)	5 (4.3)	0	1 (2.1)	19 (2.5)
sGC and PGI2	1 (0.2)	0	0	0	1 (0.1)
Dual therapy without PGI2	266 (48.5)	60 (51.3)	20 (44.4)	31 (64.6)	377 (49.7)
ERA and PDE5i	230 (41.9)	48 (41.0)	17 (37.8)	30 (62.5)	325 (42.8)
ERA and sGC	36 (6.6)	12 (10.3)	3 (6.7)	1 (2.1)	52 (6.9)
Triple therapy	47 (8.6)	9 (7.7)	4 (8.9)	4 (8.3)	64 (8.4)
ERA, PDE5i, and PGI2	45 (8.2)	8 (6.8)	4 (8.9)	3 (6.3)	60 (7.9)
ERA, PGI2, and sGC	2 (0.4)	1 (0.9)	0	1 (2.1)	4 (0.5)

*ERA* endothelin receptor antagonist, *PAH* pulmonary arterial hypertension, *PDE5i* phosphodiesterase-5 inhibitor, *PGI2* prostaglandin I2 (prostacyclin), *sGC* soluble guanylate cyclase stimulator

^a^ Other group: Asian (*n* = 26), Native Hawaiian/Pacific Islander (*n* = 3), American Indian/Alaska Native (*n* = 2), another race/ethnicity (*n* = 10), and unknown race/ethnicity (*n* = 7)

### Selexipag Dosing and Titration

In total, 387 (51.0%) participants had started selexipag ≤ 60 days before enrollment (i.e., newly initiated) and 372 (49.0%) participants had started selexipag > 60 days before enrollment (i.e., previously initiated) (Table 3). The median duration of selexipag titration ranged from 8.0 to 8.3 weeks across each of the racial/ethnic groups (Table S1 in Online Resource). The median twice-daily individualized dose was 1,100 μg overall, 1,200 μg in non-Hispanic White and Hispanic participants, and 1,000 μg in Black/African American participants (Table S1 in Online Resource). In total, 31.9% reached the maximum individualized dose of 1,600 µg twice daily: 31.7% of non-Hispanic White participants, 35.0% of Black/African American participants, and 37.8% of Hispanic participants.

**Table 3:** Selexipag discontinuation and transition to prostanoid, according to race/ethnicity

	All enrolled participants (*N* = 759)				
Parameter, *n* (%)	Non-Hispanic White (*n*=549)	Black/African American (*n*= 117)	Hispanic (*n*= 45)	Other^a^ (*n*= 48)	Overall (*N* = 759)
Discontinued selexipag	192 (35.0)	38 (32.5)	16 (35.6)	6 (12.5)	252 (33.2)
Reason for selexipag discontinuation					
Due to AE	144 (26.2)	28 (23.9)	12 (26.7)	5 (10.4)	189 (24.9)
Related to PAH progression	85 (15.5)	13 (11.1)	9 (20.0)	3 (6.3)	110 (14.5)
Related to selexipag	42 (7.7)	8 (6.8)	1 (2.2)	2 (4.2)	53 (7.0)
Unknown relationship to PAH progression or selexipag	17 (3.1)	7 (6.0)	2 (4.4)	0	26 (3.4)
Transitioned to prostanoid	74 (13.5)	11 (9.4)	8 (17.8)	2 (4.2)	95 (12.5)
	Newly initiated participants (*N* = 387)^b^				
	Non-Hispanic White (*n*= 268)	Black/African American (*n*= 65)	Hispanic (*n*= 21)	Other^a^ (*n*= 33)	Overall (*N* = 387)
Discontinued selexipag	114 (42.5)	27 (41.5)	9 (42.9)	4 (12.1)	154 (39.8)
Reason for selexipag discontinuation					
Due to AE	89 (33.2)	19 (29.2)	6 (28.6)	3 (9.1)	117 (30.2)
Related to PAH progression	43 (16.0)	9 (13.8)	4 (19.0)	1 (3.0)	57 (14.7)
Related to selexipag	34 (12.7)	6 (9.2)	1 (4.8)	2 (6.1)	43 (11.1)
Unknown relationship to PAH progression or selexipag	12 (4.5)	4 (6.2)	1 (4.8)	0	17 (4.4)
Transitioned to prostanoid	40 (14.9)	6 (9.2)	3 (14.3)	0	49 (12.7)
	Previously initiated participants (*N* = 372)^c^				
	Non-Hispanic White (*n*= 281)	Black/African American (*n*= 52)	Hispanic (*n*= 24)	Other^a^ (*n*= 15)	Overall (*N* = 372)
Discontinued selexipag	78 (27.8)	11 (21.2)	7 (29.2)	2 (13.3)	98 (26.3)
Reason for selexipag discontinuation					
Due to AE	55 (19.6)	9 (17.3)	6 (25.0)	2 (13.3)	72 (19.4)
Related to PAH progression	42 (14.9)	4 (7.7)	5 (20.8)	2 (13.3)	53 (14.2)
Related to selexipag	8 (2.8)	2 (3.8)	0	0	10 (2.7)
Unknown relationship to PAH progression or selexipag	5 (1.8)	3 (5.8)	1 (4.2)	0	9 (2.4)
Transitioned to prostanoid	34 (12.1)	5 (9.6)	5 (20.8)	2 (13.3)	46 (12.4)

*AE* adverse event, *PAH* pulmonary arterial hypertension

^a^ Other group: Asian (*n* = 26), Native Hawaiian/Pacific Islander (*n* = 3), American Indian/Alaska Native (*n* = 2), another race/ethnicity (*n* = 10), and unknown race/ethnicity (*n* = 7)

^b^ Newly initiated patients are defined as having enrolled within 60 days of selexipag initiation

^c^ Previously initiated patients are defined as having enrolled more than 60 days after selexipag initiation

At the end of the 18-month follow-up, approximately two-thirds (66.8%) of participants were still receiving selexipag (Table 3). Overall, 252 (33.2%) participants discontinued selexipag (2 [0.3%] due to death, 189 [24.9%] due to an AE, and 61 [8.0%] due to a reason other than an AE) and 95 (12.5%) participants transitioned to prostanoid. Of participants who discontinued due to an AE, 110 (14.5%) discontinued due to an AE related to PAH progression, 53 (7.0%) due to an AE related to selexipag, and 26 (3.4%) due to an unknown AE (Table 3). Discontinuations resulting from selexipag-related AEs were low for all groups, ranging from 1 (2.2%) Hispanic participant to 42 (7.7%) non-Hispanic White participants. Overall rates of selexipag discontinuations were similar (33.2%–35.6%) across race/ethnicity groups, although there were some differences between groups in reasons for discontinuation. In the Hispanic group, 20.0% of participants discontinued selexipag due to an AE related to PAH progression, compared with 11.1% in the Black/African American group. There were two deaths, one in a non-Hispanic White participant and one in a Black/African American participant.

### Time to First All-cause Hospitalization and Death

**Fig. 4:**
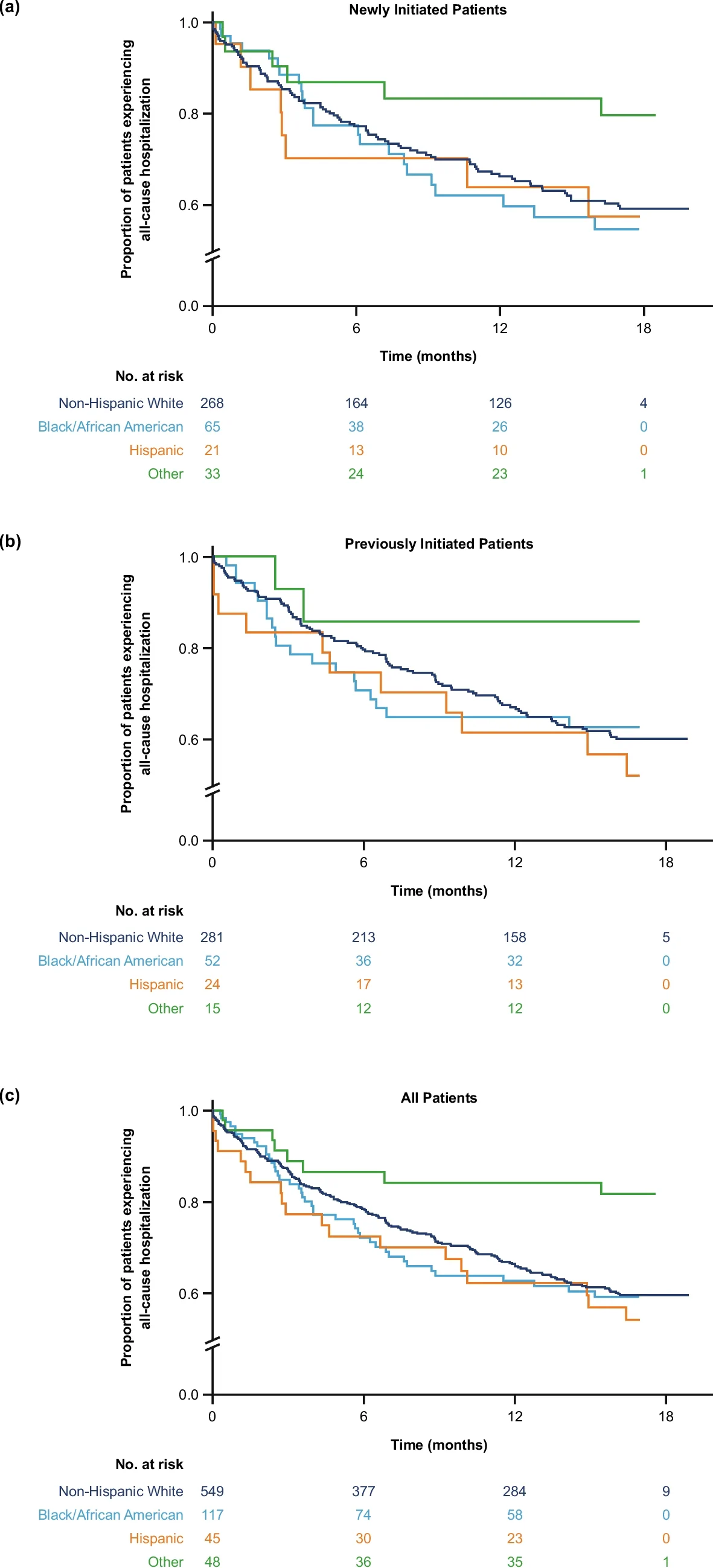
Time to first all-cause hospitalization by race/ethnicity in (**a**) newly initiated patients, (**b**) previously initiated patients, and (**c**) all enrolled patients

In total, 39.4% of patients with PAH experienced at least one hospitalization over 18 months’ follow-up. Time to first hospitalization by race/ethnicity is shown in Fig. 4a–c. The risk of hospitalization was similar between newly and previously initiated participants across all race/ethnicity groups. At 18 months, survival was 88.4% in non-Hispanic White patients, 88.2% in Black/African American patients, and 93.1% in Hispanic patients, with no difference between the groups in risk of death relative to non-Hispanic White patients. The hazard ratio for survival was 0.977 (95% CI 0.560–1.704) for Black/African American and 2.162 (95% CI 0.681–6.867) for Hispanic patients.

## Discussion

The SPHERE registry is the largest body of real-world evidence from a representative sample of patients with PAH treated with selexipag in US clinics. Previous analyses of SPHERE data have highlighted a disconnect between guideline recommendations for PAH diagnosis and treatment and actual clinical practice, including a potentially less aggressive approach to risk assessment and treatment in routine care that culminates in late selexipag initiation [5]. In the current analysis, we examined differences in patient characteristics, selexipag treatment, and outcomes across racial/ethnic groups.

Historically, racial/ethnic groups have been under-represented in major PAH clinical trials and PAH registries [7, 15]. Most major PAH trials have recruited < 20% of patients from minority racial/ethnic groups [7]. In the Phase 3 PATENT-1 study, which investigated the efficacy and safety of riociguat in PAH, only 1.4% of the study population was Black [16]. Similarly, in the Phase 3b TRITON study, which investigated the efficacy and safety of initial triple therapy with macitentan, tadalafil, and selexipag versus initial double therapy with macitentan and tadalafil in newly diagnosed patients with PAH, Black/African American patients accounted for only 4.0% of the study population [17]. Data from the US 2022 Census indicates that 13.6% of the population were Black/African American, 19.1% Hispanic, and 6.3% Asian [18]. In the more recent Phase 3 GRIPHON study, which investigated the safety and efficacy of selexipag in patients with PAH [6], Black/African American patients accounted for 13.5% and Hispanic patients for 14.8% of the study population based in the US [Data on file. Actelion Pharmaceuticals US, Inc. EDMS-RIM-259928; 2014].

Minority groups generally have greater representation in real-world registries than randomized clinical trials. In total, 15.7% of the PAH study population in the OPUS registry were Black/African American*,* but other non-White populations accounted for only 7.0% of patients [19]. In the REVEAL registry, 12.2% of patients with PAH were Black/African American, 8.9% were Hispanic, and 3.3% were Asian/Pacific Islander [20]; however, in the United States Pulmonary Hypertension Scientific Registry, only 7.4% were Black/African American and 9.6% were Hispanic [21]. In SPHERE, the proportion of Black/African Americans (15.4%) among participants treated with selexipag in routine clinical practice was similar to the overall US population; however, only 5.9% of participants were Hispanic and 3.4% were Asian. We noted regional variation in the race/ethnicity of patients with PAH in SPHERE; this has also been reported in the Pulmonary Hypertension Association Registry [22]. While this regional variation partly reflects the racial/ethnic diversity of the United States [23], our findings underscore the ongoing need to understand local and cultural barriers to equitable PAH care, as well as recruitment and enrollment of racial/ethnic minority populations in PAH clinical trials and observational registries. Notably, SPHERE is a multicenter drug registry enrolling across the US that includes only patients receiving selexipag; the under-representation of Hispanic and Asian populations might therefore reflect lack of opportunity for escalation to selexipag therapy in these patient groups.

We observed differences in the distribution of PAH subtypes between racial/ethnic groups of selexipag-treated participants in the SPHERE registry. As shown in previous observational studies [9, 10], Black/African American participants were most likely to have CTD-associated PAH. Previously published data from the PAH Biobank registry suggest that Black/African American patients with PAH are 2.5 times more likely to have a diagnosis of CTD-associated PAH compared with non-Hispanic White and Hispanic patients [10]. Similar to the PAH Biobank registry, we found higher rates of CTD-associated PAH among Black/African American participants compared with non-Hispanic White participants. Additionally, there were higher rates of CTD-associated PAH among the Hispanic population compared with the non-Hispanic White population. Hispanic participants were also most likely to have CHD-associated PAH and portal hypertension, in line with previous reports [9, 10], as well as drug/toxin-induced PAH. This latter finding might be related to geographic location, as most Hispanic participants in SPHERE resided in the Western and Southwestern regions, which have been identified as having the highest rates of methamphetamine-induced PAH in the United States [24].

The SPHERE registry did not collect data on the insurance status, socioeconomic status, or residential ZIP code of participants. Although this limitation in the current data set precludes a definitive assessment, our findings do suggest possible disparities associated with race/ethnicity in establishing a PAH diagnosis and/or access to PAH therapies. Data from REVEAL have previously shown that race/ethnicity is not associated with delayed diagnosis [25]. However, in SPHERE, we observed differences between racial/ethnic groups in age at PAH diagnosis, disease duration prior to selexipag initiation, and disease severity at selexipag initiation. There were also differences in the prevalence of comorbid conditions; for example, Black/African American participants reported the highest rates of renal failure (21.4%), type 2 diabetes (35.0%), and hypertension (65.8%), whereas Hispanic participants had the lowest rates of these conditions (4.4%, 17.8%, and 48.9%, respectively). The presence of comorbidities has been shown to delay diagnosis and negatively impact outcomes in PAH [26–28].

Our data show the youngest age at PAH diagnosis was in Hispanic participants. This could be a consequence of higher rates of CHD-associated and drug/toxin-induced PAH subtypes in the Hispanic group, which may be diagnosed earlier. Hispanic participants also had the lowest rate of treatment with concomitant PAH therapies prior to selexipag initiation and experienced the longest duration between diagnosis and selexipag initiation, although they also had less severe disease at selexipag initiation. Data from the PAH Biobank showed that Hispanic patients with PAH are less likely to be treated with PAH-specific therapies than non-Hispanic White patients [10]. Differences in social determinants of health and access, and other elements, such as implicit bias, may underlie some of these disparities [11, 15, 29, 30]. Access to specialist care centers might also play a role; most Hispanic participants in SPHERE resided in the Southwestern and Western regions, where there are relatively few specialist centers accredited by the Pulmonary Hypertension Association [31]. Interestingly, we noted that Hispanic patients had higher rates of PAH progression than other groups, perhaps related to the increased length of time between diagnosis and treatment initiation in this group.

We found that prescribing patterns in SPHERE were consistent across race/ethnicity groups. Approximately one-third of participants discontinued selexipag, with no difference between groups. Only a few participants discontinued due to a selexipag-related AE, again with no difference between race/ethnicity groups. Discontinuation of selexipag due to PAH progression was highest among Hispanic participants and lowest among Black/African American participants. Both the risk of hospitalization and survival rates were similar across race/ethnicity groups. Overall, the data suggest that, despite differences in baseline characteristics and comorbidities, selexipag treatment patterns and outcomes in routine clinical practice are broadly similar, regardless of race/ethnicity.

Our findings should be interpreted in the context of the limitations of the SPHERE study. This was an observational study, it had limited data in terms of exercise capacity and risk profiles on selexipag, and it was subject to enrollment biases and missing/incomplete data inherent to studies of this type. Racial/ethnic disparities are a result of complex interactions between patient and health system (i.e., socioeconomic status, environment, and implicit bias/discrimination) factors [7]. Underlying differences in patient and health system factors and/or bias in enrollment may contribute to differences in outcomes observed across race/ethnicity groups in this analysis. Data were not collected on insurance status, education level, or income, precluding adjustment for these social determinants of health. Furthermore, our analyses focused largely on three racial/ethnic groups (i.e., non-Hispanic White, Black/African American, and Hispanic) because of the low sample size of smaller minority populations, such as Asian American/Pacific Islanders, who represent the fastest growing ethnic population in the United States [32].

## Conclusion

This US-based PAH registry study included large numbers of racial/ethnic minority populations that are typically under-represented in randomized clinical trials, particularly Black/African Americans. However, Hispanic and Asian patients were still largely under-represented, underscoring the need to understand barriers to access to care and timely treatment escalation in these groups. Nevertheless, despite diversity and differences in demographics and comorbid conditions, treatment patterns and outcomes (i.e., hospitalization rates and survival) observed in routine clinical practice were similar for all the racial/ethnic groups. Selexipag was prescribed as part of combination therapy for PAH regardless of differing demographics, comorbidities, and disease severity across the groups. These findings can help guide treatment decisions in populations that may be under-represented in clinical trials.

## Supplementary Information

Below is the link to the electronic supplementary material.

Supplementary file1 (DOCX 201 KB)

## Data Availability

The data sharing policy of the sponsor is available at https://www.janssen.com/clinical-trials/transparency. Although these data that support the findings of this study are not currently publicly available for sharing due to privacy or ethical restrictions, requests for sharing can be sent to the corresponding author and will be evaluated on an individual basis.
