# Supplementary material for: Characteristics of Patients with Pulmonary Arterial Hypertension Receiving Selexipag in the SPHERE Registry by Race and Ethnicity
Source: J Racial Ethn Health Disparities. 2025 Aug 4;13(5):4283–95. doi: 10.1007/s40615-025-02579-3 (PMC13546152; doi:10.1007/s40615-025-02579-3)
Supplement: Supplementary file 1 [file 40615_2025_2579_MOESM1_ESM.docx]

**SUPPLEMENTARY INFORMATION**

***Journal of Racial and Ethnic Health Disparities***

**Characteristics of Patients with Pulmonary Arterial Hypertension Receiving Selexipag in the SPHERE Registry by Race and Ethnicity**

**Harrison W. Farber, Murali M. Chakinala, Anna R. Hemnes, Kelly M. Chin, Kristin B. Highland, Vallerie V. McLaughlin, Lana D. Melendres-Groves, Michelle Cho, Gurinderpal Doad, Elham Fatehi, Michelle Han, Mohammad Rahman, Paul Strachan, Tobore Tobore, Nick H. Kim**

**Corresponding author:** Harrison W. Farber, email: harrison.farber1@tuftsmedicine.org

**Table S1** Selexipag dosing regimen and titration schemes in patients with PAH, according to race/ethnicity

| **Parameter** | **Non-Hispanic White  (n = 549)** | **Black/African American  (n = 117)** | **Hispanic  (n = 45)** | **Other^a^ (n = 48)** | **Overall  (N = 759)** |
| --- | --- | --- | --- | --- | --- |
| Duration of the titration phase, weeks |  |  |  |  |  |
| Median (IQR) | 8.1 (5.1–10.9) | 8.0 (5.0–11.0) | 8.3 (4.6–11.1) | 8.1 (5.3–13.1) | 8.1 (5.0–11.1) |
| Minimum, maximum | 1.1, 26.9 | 1.1, 26.4 | 1.1, 22.3 | 1.1, 25.7 | 1.1, 26.9 |
| Uptitration speed (µg twice daily/week), n (%) |  |  |  |  |  |
| < 200 | 490 (89.3) | 102 (87.2) | 39 (86.7) | 40 (83.3) | 671 (88.4) |
| ≥ 200 | 59 (10.7) | 15 (12.8) | 6 (13.3) | 8 (16.7) | 88 (11.6) |
| Selexipag maintenance dose, μg twice daily |  |  |  |  |  |
| n | 527 | 111 | 45 | 46 | 729 |
| Median (IQR) | 1,200 (600–1,600) | 1,000 (600–1,600) | 1,200 (600–1,600) | 1,000 (600–1,400) | 1,100 (600–1,600) |
| Minimum, maximum | 100, 3,200 | 100, 1,600 | 100, 1,600 | 100, 1,600 | 100, 3,200 |

IQR, interquartile range; PAH, pulmonary arterial hypertension

^a^ Other group: Asian (n = 26), Native Hawaiian/Pacific Islander (n = 3), American Indian/Alaska Native (n = 2), another race/ethnicity (n = 10), and unknown race/ethnicity (n = 7)

**Fig. S1** Regional variability in race/ethnicity among patients with PAH in the SPHERE registry

PAH = pulmonary arterial hypertension; SPHERE = SelexiPag: tHe usErs dRug rEgistry
